# Retraction Note: Doubled haploid production in alfalfa (*Medicago sativa L.*) through isolated microspore culture

**DOI:** 10.1038/s41598-019-48912-9

**Published:** 2019-09-13

**Authors:** Dengxia Yi, Jifeng Sun, Yanbin Su, Zongyong Tong, Tiejun Zhang, Zan Wang

**Affiliations:** 1 Institute of Animal Science, Chinese Academy of Agricultural Sciences, Beijing, 100193 China; 2 Weifang Academy of Agricultural Science, Weifang, Shandong 261071 China; 3 Beijing Zhongnong Futong Horticulture Corporation Limited, Beijing, 100193 China

Retraction of: *Scientific Reports* 10.1038/s41598-019-45946-x, published online 01 July 2019

This article has been retracted by the authors because it was submitted without the knowledge or consent of the last author.

All authors agreed to the retraction.

